# Insights into the genome structure of four acetogenic bacteria with specific reference to the Wood–Ljungdahl pathway

**DOI:** 10.1002/mbo3.938

**Published:** 2019-10-01

**Authors:** Alfonso Esposito, Sabrina Tamburini, Luca Triboli, Luca Ambrosino, Maria Luisa Chiusano, Olivier Jousson

**Affiliations:** ^1^ Department of Cellular, Computational and Integrative Biology—CIBIO University of Trento Trento Italy; ^2^ Department of Genetics and Genomic Sciences Icahn School of Medicine at Mount Sinai NY USA; ^3^ Research Infrastructures for Marine Biological Resources (RIMAR) Stazione Zoologica Anton Dohrn Naples Italy; ^4^ Department of Agricultural sciences University of Naples “Federico II” Portici Italy

**Keywords:** Acetogens, Comparative genomics, NGS, Wood–Ljungdahl pathway

## Abstract

Acetogenic bacteria are obligate anaerobes with the ability of converting carbon dioxide and other one‐carbon substrates into acetate through the Wood–Ljungdahl (WL) pathway. These substrates are becoming increasingly important feedstock in industrial microbiology. The main potential industrial application of acetogenic bacteria is the production of metabolites that constitute renewable energy sources (biofuel); such bacteria are of particular interest for this purpose thanks to their low energy requirements for large‐scale cultivation. Here, we report new genome sequences for four species, three of them are reported for the first time, namely *Acetobacterium paludosum* DSM 8237, *Acetobacterium tundrae* DSM 917, *Acetobacterium bakii* DSM 8239, and *Alkalibaculum bacchi* DSM 221123. We performed a comparative genomic analysis focused on the WL pathway's genes and their encoded proteins, using *Acetobacterium woodii* as a reference genome. The Average Nucleotide Identity (ANI) values ranged from 70% to 95% over an alignment length of 5.4–6.5 Mbp. The core genome consisted of 363 genes, whereas the number of unique genes in a single genome ranged from 486 in *A. tundrae* to 2360 in *A.bacchi*. No significant rearrangements were detected in the gene order for the Wood–Ljungdahl pathway however, two species showed variations in genes involved in formate metabolism: *A. paludosum* harbor two copies of *fhs1*, and *A. bakii* a truncated *fdhF1*. The analysis of protein networks highlighted the expansion of protein orthologues in *A. woodii* compared to *A. bacchi*, whereas protein networks involved in the WL pathway were more conserved. This study has increased our understanding on the evolution of the WL pathway in acetogenic bacteria.

## Graphical abstract

Acetogenic bacteria are able to convert one‐carbon substrates into acetate through the Wood‐Ljungdahl (WL) pathway. We report a comparative genomic analysis of four acetogenic species with a focus on the genes encoding enzymes of the WL pathway. We found variations in the organization of WL pathway gene clusters and an expansion of protein orthologues.

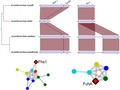

## 1 INTRODUCTION

Acetogenic bacteria, or acetogens, are obligate anaerobes converting one‐carbon substrates, such as carbon dioxide, formate, methyl groups, or carbon monoxide into acetate using molecular hydrogen as electron donor through the Wood–Ljungdahl (WL) pathway, a process known as acetogenesis (Ragsdale & Pierce, 2008). Acetogenesis was first described in the early '30 and has been extensively studied in Clostridia (Drake, 1994). The WL pathway was considered for a long time to be a specific trait of species belonging primarily to the Firmicutes (Ragsdale & Pierce, 2008), but a number of recent studies have shown that this pathway is far more spread in the microbial tree of life than previously thought (Adam, Borrel, & Gribaldo, 2018; Borrel, Adam, & Gribaldo, 2016; Graber & Breznak, 2004; Hug et al., 2013; Strous et al., 2006). Acetogenic species have been found in the archaeal kingdom, although most Archaea produce methane instead of acetate as end product (Borrel et al., 2016), in Chloroflexi (Hug et al., 2013), Spirochetes (Graber & Breznak, 2004), and Planctomycetes (Berg, 2011; Strous et al., 2006).

Due to its low ATP requirement, the WL pathway can be found in prokaryotes adapted to conditions that approach the thermodynamic limits of life (Schuchmann and Mueller, 2014). In addition, comparative genomic analyses of extant microbial taxa revealed that the predicted last common universal ancestor possessed the WL pathway (Adam et al., 2018; Weiss et al., 2016). It is thus conceivable that the WL pathway represented an efficient way to produce energy in the early Earth environment before the great oxidation event, that is the enrichment of oxygen in the early earth atmosphere as a consequence of the emergence of organisms able to perform oxygenic photosynthesis (Poehlein et al., 2012; Weiss et al., 2016). The main advantages of the WL pathway include the following: its versatility; it can be coupled to methanogenesis or to energy conservation via generation of electrochemical gradients; its modularity, since some species utilize partial WL pathways to channel electrons produced during fermentation to CO_2_; its flexibility, as several organisms use different coenzymes and/or electron carriers, and in some cases the WL pathway is reversed (e.g., it generates molecular hydrogen and carbon dioxide from acetate for energy production (Schuchmann & Mueller, 2016).

**Figure 1:**
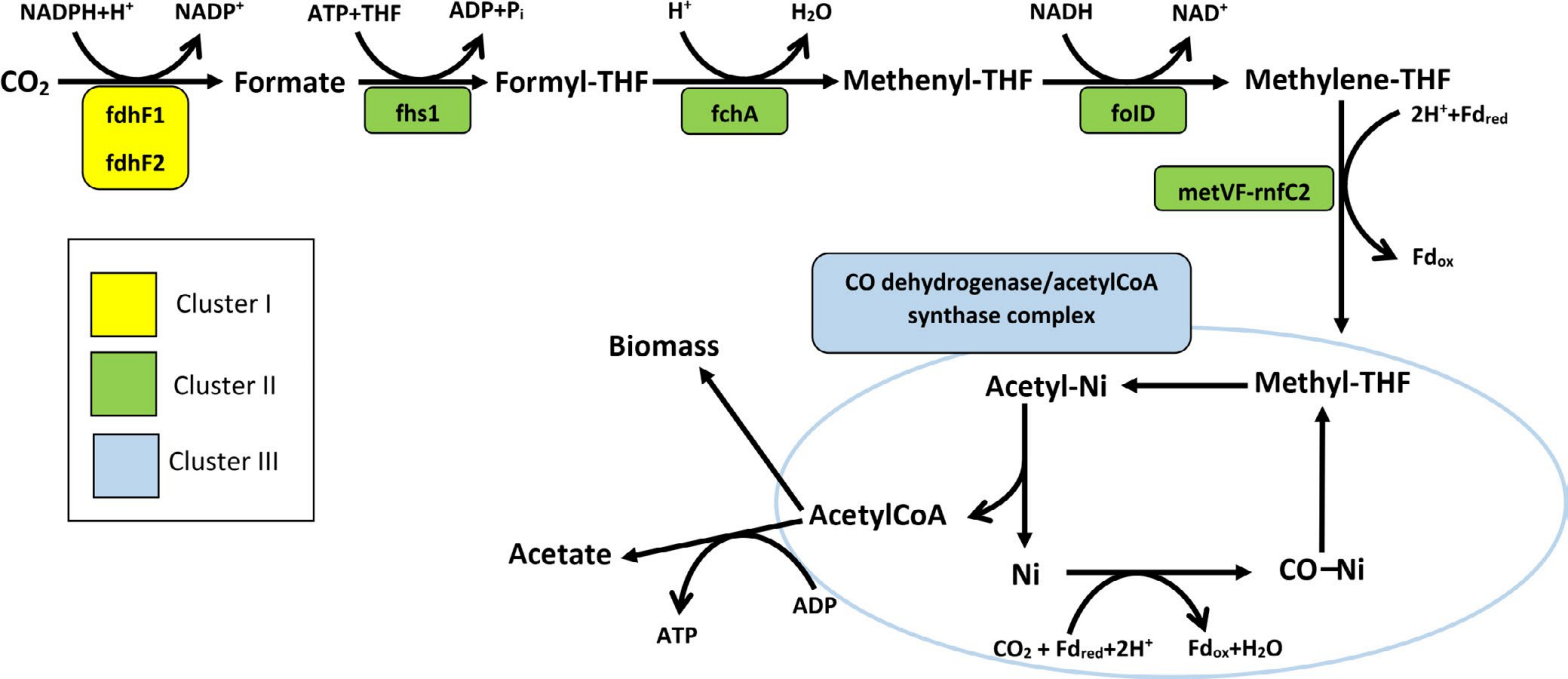
Graphic depiction of the Wood–Ljungdahl pathway including the genes involved in each step of the pathway. Colors represent the gene clusters; THF: tetrahydrofolate; fdhF1 and 2: formate dehydrogenase 1 and 2; fhs1: formyl‐THF synthetase; fchA:methenyl‐THF cyclohydrolase, folD: methylene‐THF dehydrogenase; metVF: methylene‐THF reductase; rnfC2: rnfC‐like protein. Redrawn from Poehlein et al. (2012)

There is a growing interest toward acetogens, as they can be used as biocatalyst for the conversion of synthesis gas (a mixture of H_2_ and CO and/or CO_2_) into fuels or chemicals with low energy supply (Bengelsdorf et al., 2016; Cavicchioli et al., 2011; Shin et al., 2018). The genome structure and encoded functions of the members of the genus *Acetobacterium* (Balch, Schoberth, Tanner, & Wolfe, 1977), are still not very well understood. The genes involved in the WL pathway of *Acetobacterium woodi* are divided into three clusters (Poehlein et al., 2012). Each of them consists of 6 to 10 syntenic genes, with their products orchestrating a specific phase of the WL pathway (Figure 1). Cluster I consists of 7 genes encoding formate dehydrogenase and accessory enzymes catalyzing the reduction of carbon dioxide to formate. Cluster II contains 6 genes, underpinning the four steps leading from formate to acetyl‐CoA. Cluster III encodes the enzymes involved in carbon fixation and production of acetate from acetyl‐CoA (Poehlein et al., 2012). Here, we report new genome sequences of four acetogenic bacteria and perform a comparative genomic analysis focused on the gene clusters and protein networks of the WL pathway.

## 2 MATERIALS AND METHODS

### 2.1 Bacterial strains

*Acetobacterium paludosum* DSM 8237, *Acetobacterium tundrae* DSM 917, *Acetobacterium bakii* DSM 8239, *Alkalibaculum bacchii* DSM 221123 were obtained from the Leibniz Institute DSMZ—German Collection of Microorganisms and Cell Cultures. The bacterial strains were grown in Difco sporulation media (DSM) under anaerobic conditions (Table 1). The three *Acetobacterium* species were grown in DSM 614 medium amended with fructose at a temperature of 22°C, while *Alkalibaculum bacchi* was grown in DSM 545 medium at a temperature of 37°C.

**Table 1:** NGS data and genome assembly statistics

	# read pairs	# contigs	N50	Tot. length	% GC
*A. bacchi* DSM 22112	553976	49	186894	3,116,598	34.71
*A. bakii* DSM 8239	786768	43	285194	4,163,517	41.21
*A. paludosum* DSM 8237	1158287	54	179628	3,691,131	40.04
*A. tundrae* DSM 9173	757003	66	154452	3,563,081	39.64

### 2.2 DNA extraction, library preparation, and sequencing

Genomic DNA was extracted using the Qiagen DNeasy Blood and Tissue kit (Hilden, Germany), according to the manufacturer's protocol for gram‐positive bacteria. Bacterial cells were harvested by centrifugation at 10,000*g* for 15 min and kept at 37°C for 1 hr with the enzymatic lysis buffer provided by the supplier. Cells were then placed at 56°C for 30 min and treated with RNase A. After column purification, DNA was eluted with 100 ml 10 mmol/L Tris/HCl, pH 8.0. Genomic DNA purity and integrity were assessed by measuring the absorbance at 260 nm (A260) and the ratio of the absorbance at 260 and 280 nm (A260/A280) with a NanoDrop ND‐1000 spectrophotometer (Thermo Scientific). Genomic DNA concentration was measured by using the Qubit fluorometer (Thermo Fisher). Libraries were prepared using the Nextera XT DNA library preparation kit (Illumina, USA) with default settings, and sequenced on an Illumina MiSeq platform.

### 2.3 Genome assembly and annotation

The quality of the reads was checked using the software fastqc (Andrews, 2010), and adaptor sequences were removed using trim_galore (Krueger, 2016). The assembly was performed with the software SPAdes version 3.8.0 (Bankevich et al., 2012), using all default parameters and the option “–careful.” After assembly, contigs shorter than 500 bp and/or with a coverage below 3 were removed. Pairwise Average Nucleotide Identity (ANI) values were calculated among the five sequenced genomes and the reference genome of *A. woodii* using the software pyani (Pritchard, Glover, Humphris, Elphinstone, & Toth, 2016). The output was visualized using the in‐house developed software DiMHepy, publicly available at https://github.com/lucaTriboli/DiMHepy.

Genomes were annotated using Prokka (Seemann, 2014), using an ad hoc database created starting from the genome of *A. woodii*. Amino acidic sequences predicted by Prokka were used as input for EggNOG mapper for prediction of functional features (Huerta‐Cepas et al., 2017). The outputs of Prokka were imported in R (R Core Team, 2012) for graphical depiction of genomic maps using the R‐package GenoPlotR (Guy, Kultima, Andersson, & Quackenbush, 2011), based on the coordinates found by Prokka. To infer the number of shared genes among the five genomes we used Roary (Page et al., 2015), leaving all default settings beside the blastp identity parameter, that was set to 60 because the comparative analysis included a species from another genus (i.e., *Alkalibaculum bacchi*). Venn diagrams, based on presence/absence of homologous genes as inferred by Roary, were drawn using the web tool of the Bioinformatics and Evolutionary Genomics Department of the University of Gent (http://bioinformatics.psb.ugent.be/webtools/Venn/).

To identify biosynthetic gene clusters for secondary metabolites, the genome sequences for each of the strains were uploaded in fasta format to the antibiotics and Secondary Metabolites Analysis SHell (antiSMASH) web server (Blin et al., 2017).

### 2.4 Prediction of orthologues and paralogues

The protein sequences for the five species were predicted by Prokka, and all‐versus‐all sequence similarity searches between the protein set of each pair of the five considered species were performed independently using the BLASTp program of the BLAST package (Camacho et al., 2009). As proposed by Rosenfeld and DeSalle (2012), a paralogy analysis may consider an E‐value threshold that maximizes the number of detectable protein families (Rosenfeld & DeSalle, 2012). Therefore, all similarity searches were initially carried out using an E‐value cutoff of 10^−3^. In order to identify orthologues, we used a python software developed by Ambrosino et al. (2018). The software accepts the output of the BLAST similarity searches as input, implementing a Bidirectional Best Hit (BBH) approach (Hughes, 2005; Huynen & Bork, 1998; Overbeek, Fonstein, D'Souza, Pusch, & Maltsev, 1999; Tatusov, Koonin, & Lipman, 1997). Such approach establishes that proteins a_i_ and b_i_, from species A and B, respectively, are the best orthologues if a_i_ is the best scored hit of b_i_, with b_i_ being the best scored hit of a_i_, in all‐versus‐all BLAST similarity searches (Hughes, 2005). For paralogy prediction, all‐versus‐all similarity searches were performed for each species using the BLASTp program.

### 2.5 Protein similarity networks

Networks of proteins based on the inferred similarity relationships were built. The network construction procedure extracted all the connected components into different separated undirected graphs by using NetworkX package (Hagberg, Schult, & Swart, 2008). Each node in the network represents a protein and each edge represents an orthology or paralogy relationship. A filtering step was introduced to select for each species only the E‐value cutoff that maximized the number of paralogue networks. The selected E‐values were e^‐10^ for *Acetobacterium woodii, A. paludosum*, *A. tundrae,* and *A. bakii*, and e^‐5^ for *Alkalibaculum bacchi*. Cytoscape software (Shannon et al., 2003) was used for the graphical visualization of the networks.

## 3 RESULTS AND DISCUSSION

### 3.1 Genome‐wide analyses reveal close similarity between *A. tundrae* and *A. paludosum*

**Figure 2:**
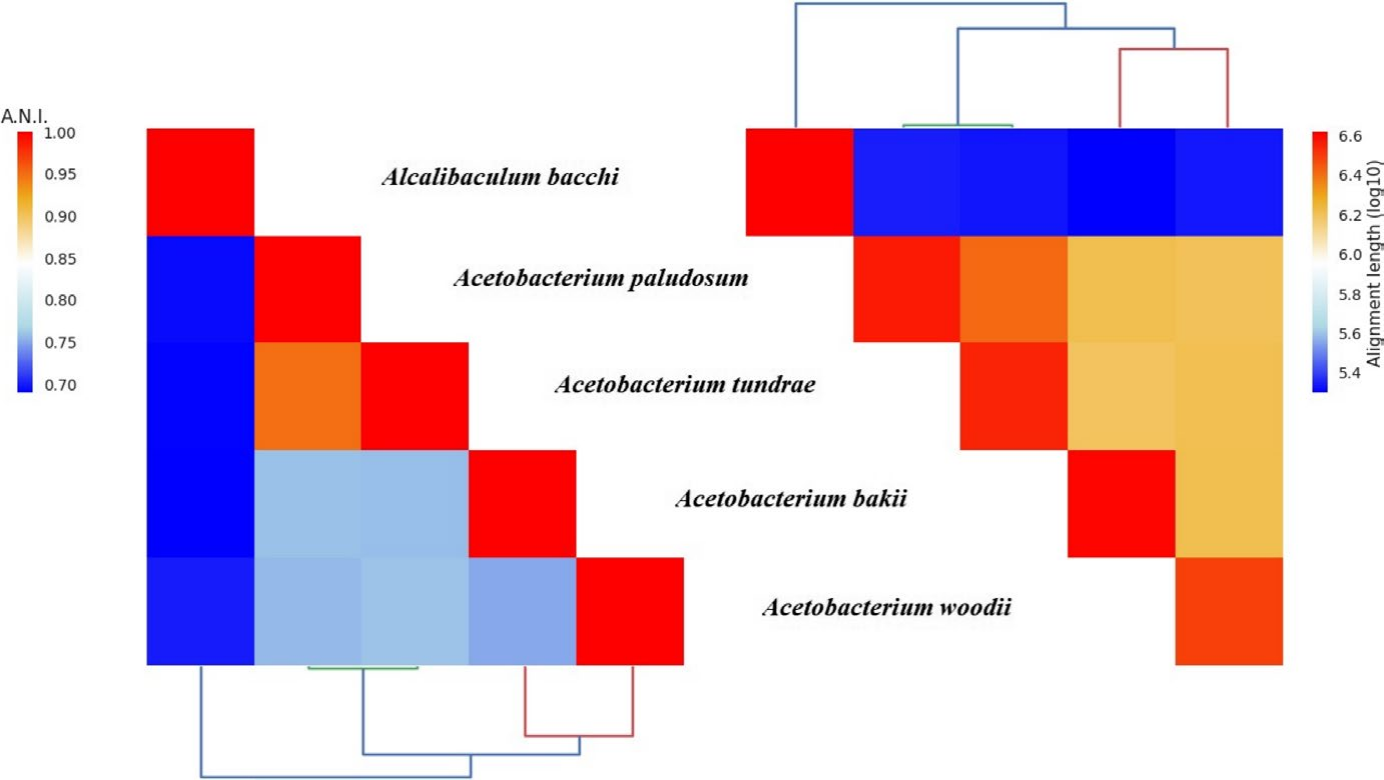
Hierarchically clustered heatmap of ANI calculated using blastn (left), and alignment length (right) between the five genomes

The number of reads per genome was on average 814.008 ± 251.751; the assembly resulted in an average number of contigs of 53 ± 9 (Table 1). Genome lengths ranged from 3.1 up to 4.1 Mbp; within the *Acetobacterium* genus the range was 3.1–3.7. The genome of *A. bacchi* was the largest one, with a size of 4.1 Mbp, an N50 ranging 186.894–285.194 with an average of 201.542 ± 57.474 (Table 1). Genome annotation statistics were consistent with the values reported in a previous pan‐genomic study focussing on 23 bacteria (22 of which belonging to the phylum Firmicutes) (Shin, Song, Jeong, & Cho, 2016). The ANI values calculated across the five genomes ranged from 70% to 95%, the alignment length ranged from 5.4 up to 6.5 Mbp. The analysis showed that *A. tundrae* and *A. paludosum* genomes had the highest ANI value (94.9%) and the largest alignment length (6.3 Mbp, Figure 2). It should be pointed out that *A. bakii* DSM 8239 was sequenced in another study (Hwang, Song, & Cho, 2015). We compared the previously sequenced genome of *A. bakii* with our data and found an ANI value of 99.76% over an alignment length of 4.12 Mb.

The ANI analysis confirms the evolutionary relationships between these species (Simankova et al., 2000), with *A. paludosum* and *A. tundrae* being most closely related within the genus *Acetobacterium* with an ANI of 95% over an alignment length of 6.4 Mbp. *Alkalibaculum bacchi* branched outside of the *Acetobacterium* group, and displayed an ANI value of 70%, over an alignment length of 5.4 Mbp.

The annotation using Prokka found on average 3,343 ± 393 coding sequences. Proteins were assigned using EggNOG mapper to 2,460 ± 221 protein families (Table 2).

**Table 2:** Genome annotation statistics, including number of CDS predicted by Prokka, antiSMASH gene clusters analysis and protein family annotation by eggNOG mapper (for *A. woodii* the analysis was done on the reference strain with acc.no. CP002987)

	Coding sequences (CDS)	Avg. # CDS per Kb	Avg. gene length	% genome containing CDS	#rRNA	#tRNA	# Protein Families	Secondary metabolites gene clusters found by antiSMASH					
								Bacteriocin/ Microcin	Terpene	NRPS	fatty acids	saccharide	others
*A.woodii* 1030	3618	0.89	951.6	85.11	16	58	2698	1	0	2	1	4	9
*A. bacchi* 22112	2860	0.92	898.7	82.48	6	55	2205	1	0	1	1	4	5
*A. bakii* 8239	3822	1.23	936.6	85.97	5	48	2740	2	1	0	1	4	8
*A. paludosum* 8237	3363	1.08	947.2	86.3	6	53	2487	2	0	0	1	3	9
*A. tundrae* 9173	3330	1.07	919.2	85.13	6	54	2411	3	0	1	1	3	10

The number of gene clusters involved in the production of secondary metabolites identified by the antiSMASH analysis was 12, 16, 15, and 18 in *A. bacchi*, *A. bakii*, *A. paludosum,* and *A. tundrae*, respectively (Table 2). A single cluster of genes for fatty acid biosynthesis per genome was found by the ClusterFinder algorithm, and this cluster was in all cases homologous to a cluster of 10 genes in *Streptococcus pneumoniae*. In the four *Acetobacterium* species, the antiSMASH analysis detected a cluster of genes involved in bacteriocin production. This cluster consisted of 7 syntenic genes homologous to a cluster of genes in *A. woodii* including two radical SAM proteins, two B12‐binding domain‐containing radical SAM protein, one HlyD family efflux transporter periplasmic adaptor subunit, one Nif11‐like leader peptide family natural product precursor, and a hypothetical protein. This gene cluster was not found in *A. bacchi*.

**Figure 3:**
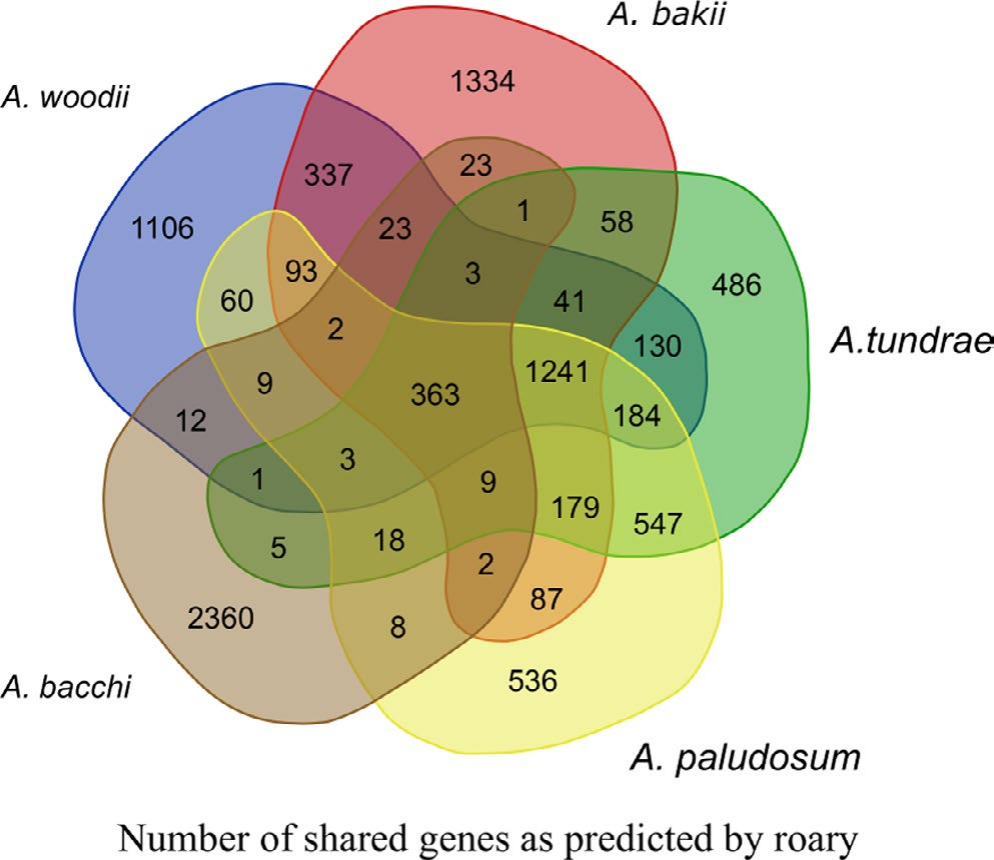
Venn diagram summarizing the number of shared and unique genes as inferred by Roary

The pangenome consisted of 9,262 genes, with a core genome of 363 genes (whose annotation is provided in Table A1), the number of core genes *Acetobacterium* spp. was 1,241. The number of unique genes into a single genome ranged from 486 to 2,360, in *A. tundrae* and *A. bacchi*, respectively (Figure 3).

### 3.2 Gene cluster organization of the WL pathway is well conserved in *Acetobacterium spp*

**Figure 4:**
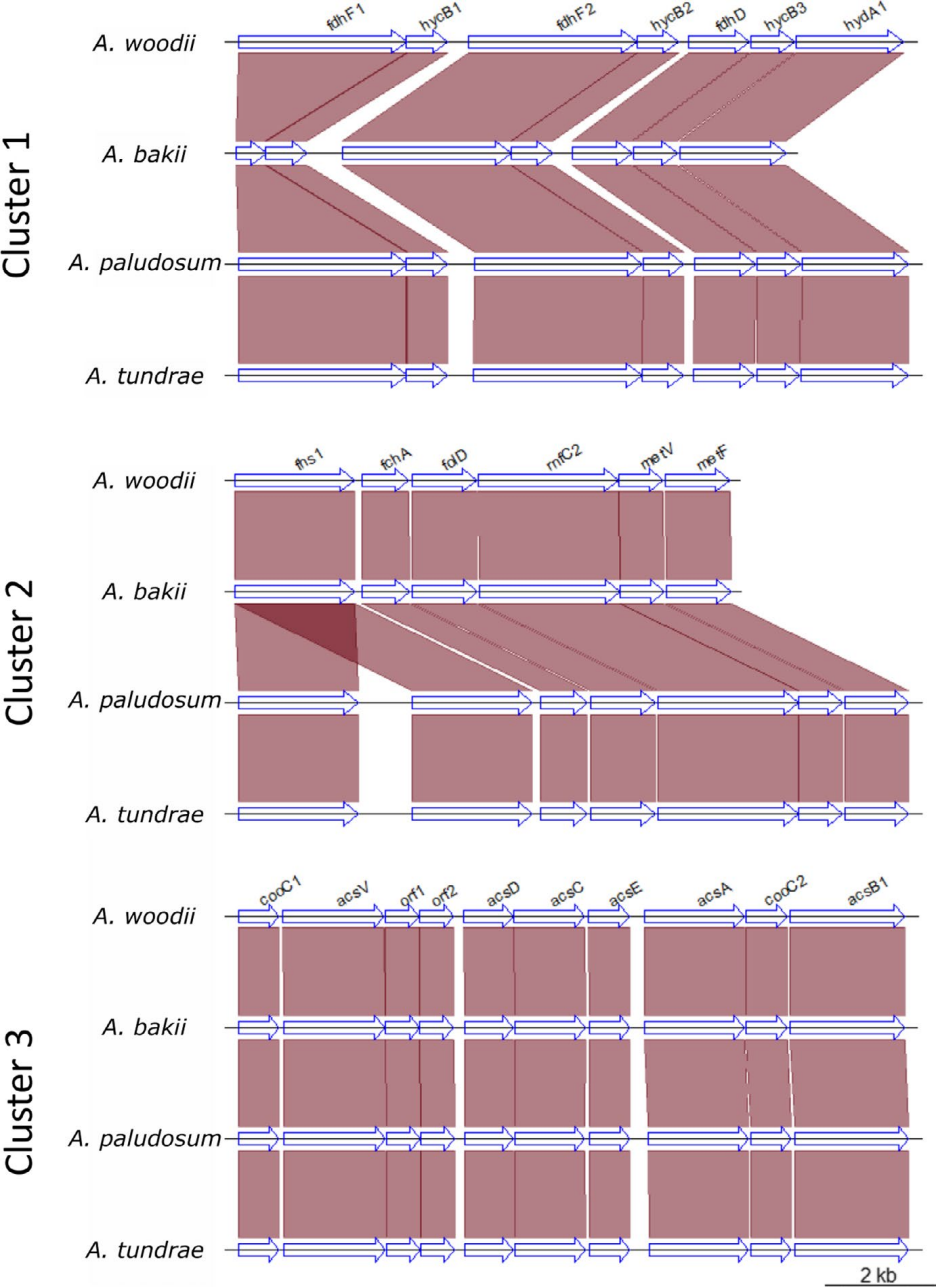
Organization of the three gene clusters in the four *Acetobacterium* genomes. Orthologues are connected with purple shades

As mentioned above, the WL pathway in *A. woodii* is encoded by three gene clusters. We examined the organization of those genes in three newly sequenced *Acetobacterium* species. The gene order was perfectly conserved (syntenic), compared with the reference strain *Acetobacterium woodii,* in the three clusters. *A. bakii* showed a truncated version of the formate dehydrogenase gene (*fdhF1*), whereas the other genes in this cluster were conserved (Figure 4). To confirm this observation, we searched the homologue of *fdhF1* in the genome of *A. bakii* deposited in NCBI, which could not be identified. Consistently, a truncated version of *fdhF1* in *A. bakii* was also found by Shin et al. (2018). In the genomes of *A. tundrae* and *A. paludosum*, the gene encoding formyl‐tetrahydrofolate synthetase (*fhs1*, from cluster II), was duplicated (Figure 4). One possible explanation for this feature could be the duplication of this specific gene as an adaptive trait. Examples of gene duplication are frequently connected to environmental adaptation (Tatusov et al., 1997), often through gene dosage (Bratlie et al., 2010; Kondrashov, 2012).

Gene cluster III presented no rearrangements in any of the four *Acetobacterium* genomes (Figure 4). Conversely, in *Alkalibaculum bacchi*, genes of the WL pathway were organized in a different way compared to the *Acetobacterium* genus, as none of the three clusters was found to be complete. Genes appeared instead to be scattered all over the bacterial chromosome (Table A2). Only the formate dehydrogenase genes (and not the accessory proteins) of cluster I were found on two separate contigs. All genes of cluster II were found, although they were split between two contigs. All but two genes of cluster III were found on the same contig, although the gene order was not maintained (Table A2).

### 3.3 Protein network analysis reveals gene expansion dynamics for WL pathway proteins

**Figure 5:**
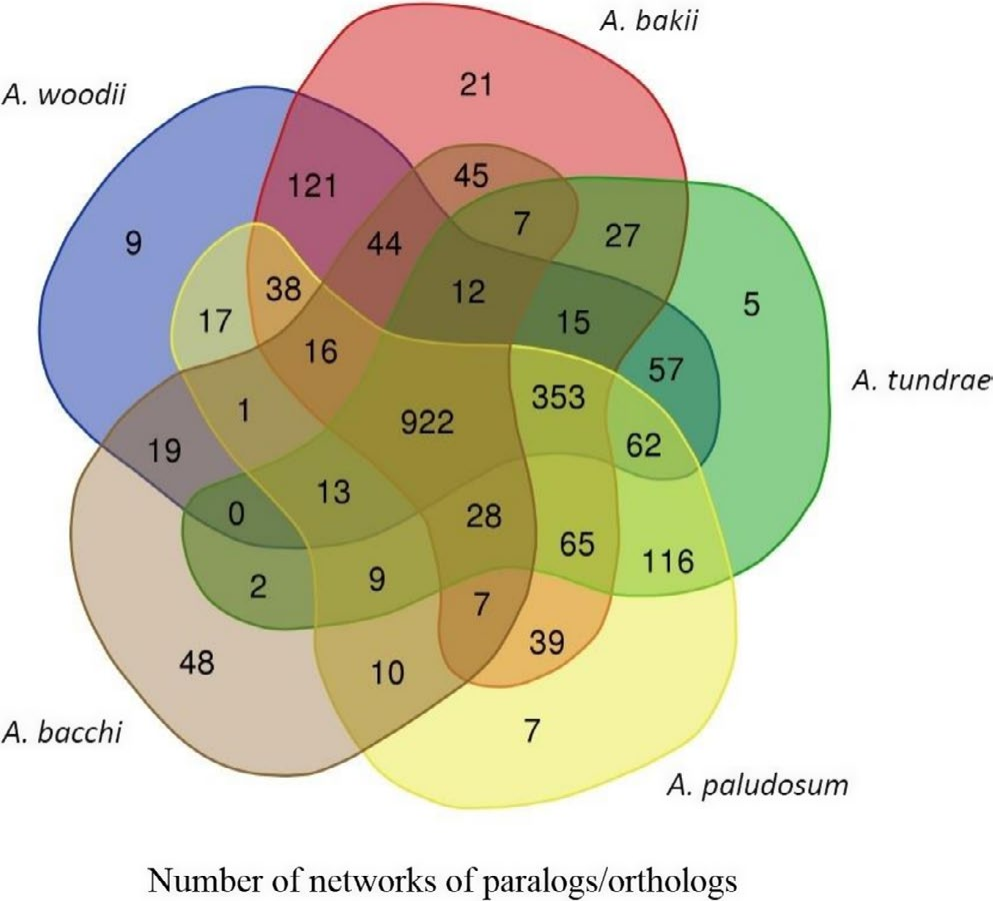
Venn diagram summarizing the number of networks that include proteins from the five considered species

The comparative analysis performed on all considered species led to the construction of networks of protein orthologues and paralogues. Prediction of orthologues between the five species was performed using a Bidirectional Best Hit (BBH) approach. Overall, 20,712 BBHs were detected. Paralogues were detected by all‐against‐all sequence similarity searches. Using as an input the predicted 20,712 orthology relationships, we considered the associated paralogues in all species, which led to the identification of a total of 2,135 distinct networks (Figure 5). A general overview of the generated networks indicates that a consistent core of networks (922) contained proteins present in all considered species, while only 9, 21, 5, 7, and 48 networks contained proteins exclusively found in *A. woodii*, *A. paludosum*, *A. tundrae*, *A. bakii,* and *A. bacchi,* respectively (Figure 5).

**Figure 6:**
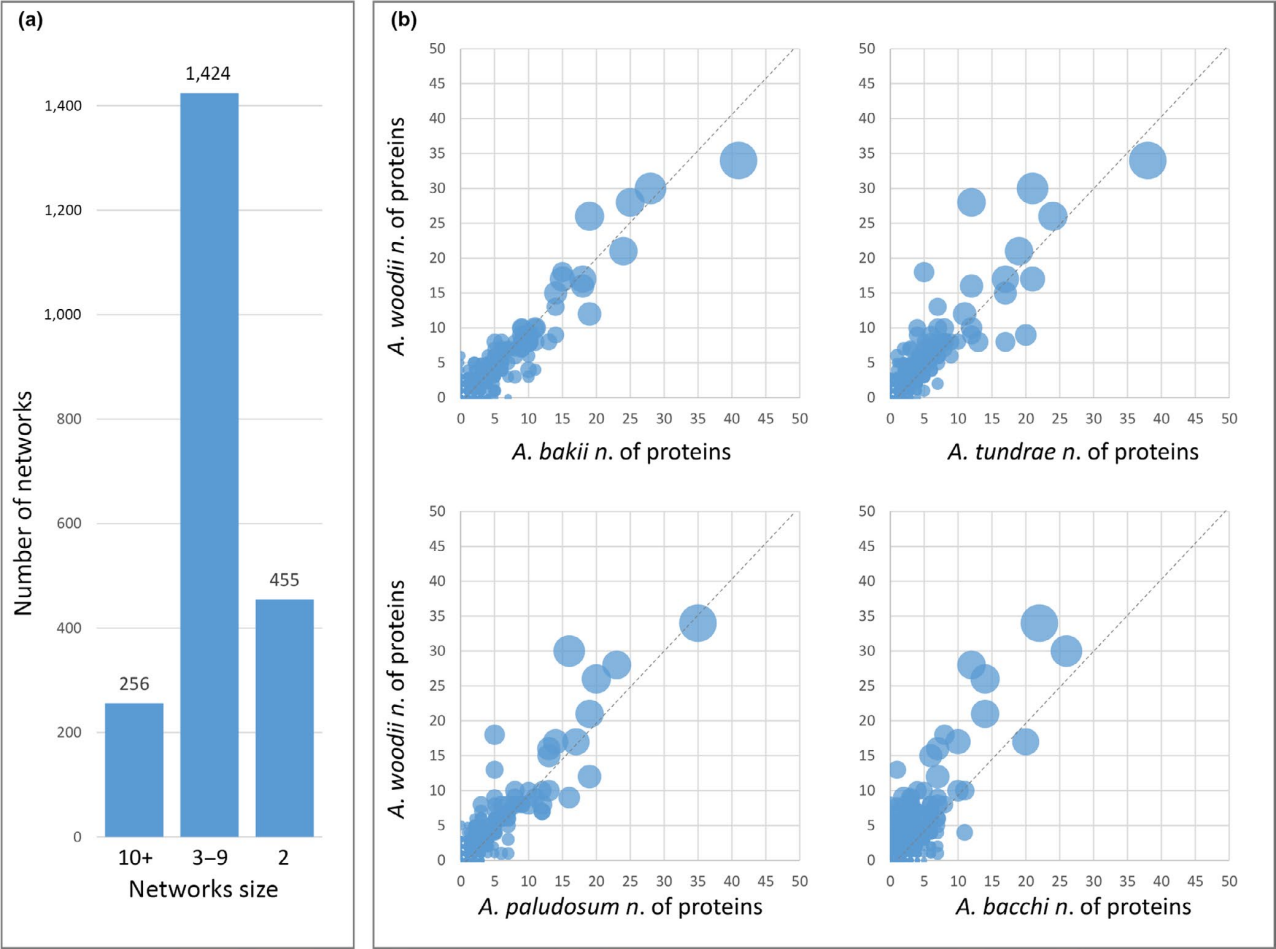
Overview of the defined protein networks highlighting the respective distribution per species. (a) Bar chart showing the number of networks classified according to their size; (b) Scatter plots showing the distribution of the networks based on the respective number of proteins from *A. woodii* compared to the other considered species. Circle diameter is proportional to the number of BBHs within each network

We then inferred gene conservation or divergence between species pairs, calculating the number of proteins per species for each network (Figure 6). We defined duplicated proteins starting exclusively from the previously detected orthologue pairs. Specifically, we defined 455 two‐protein networks connected by a single orthology relationship, 1,424 networks including 3–9 proteins, and 256 networks containing 10 or more proteins (Figure 6a). The networks distributed along a hypothetical bisector (Figure 6b), which represent the protein families that did not undergo significant changes in the number of members between species pairs. In contrast, networks that are distant from the bisector represent expansions or reductions in the number of proteins of related protein families in *A. woodii* compared to the other species. Furthermore, it is possible to infer the most conserved protein families between *A. woodii* and the other species by considering the networks with the highest number of orthologues (large circles in Figure 6).

**Figure 7:**
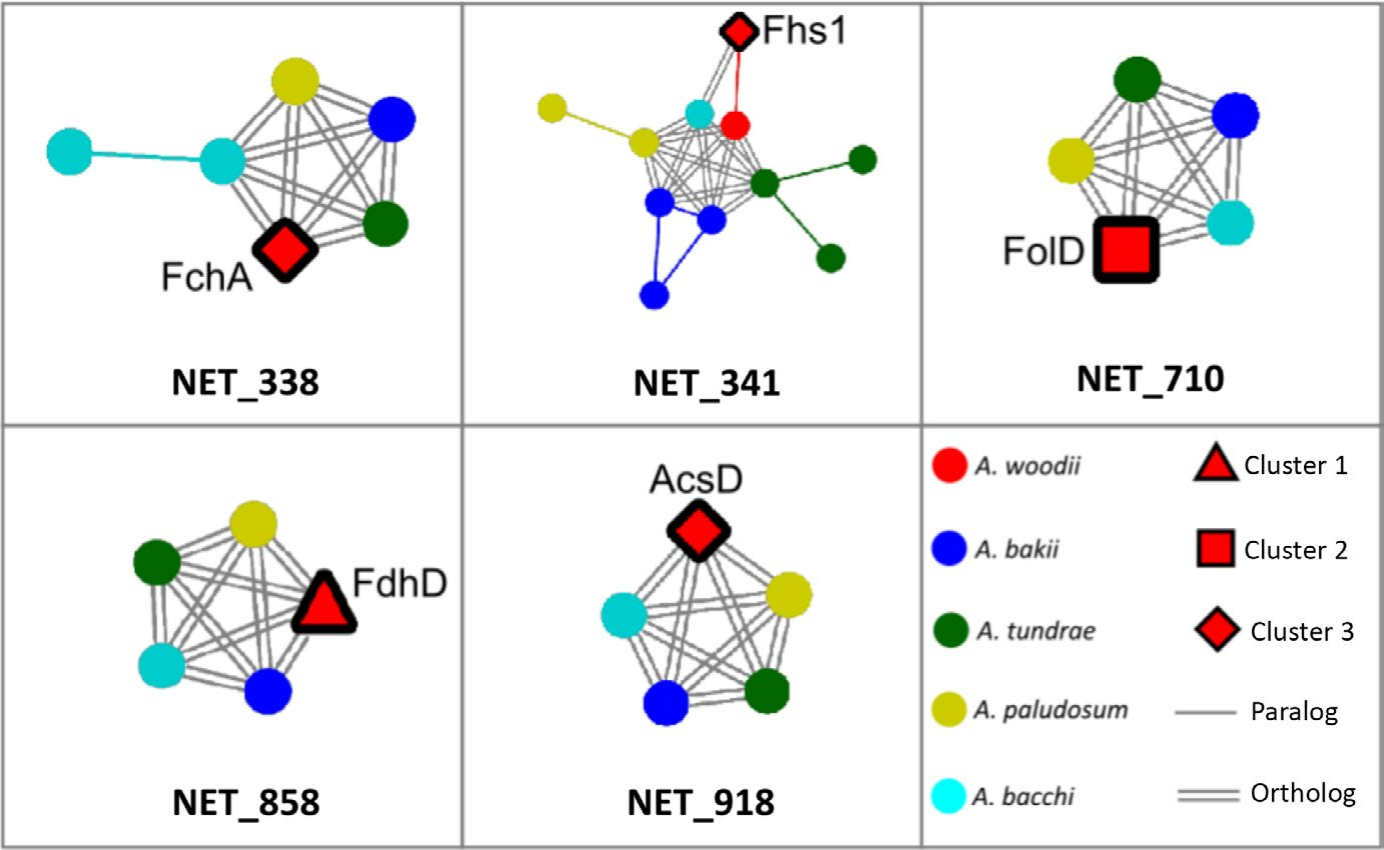
Selected networks displaying different amplification patterns in genes involved in the Wood–Ljungdahl pathway. An extended version of this figure including all proteins of the WL pathway is presented in Figure A1

**Figure A1:**
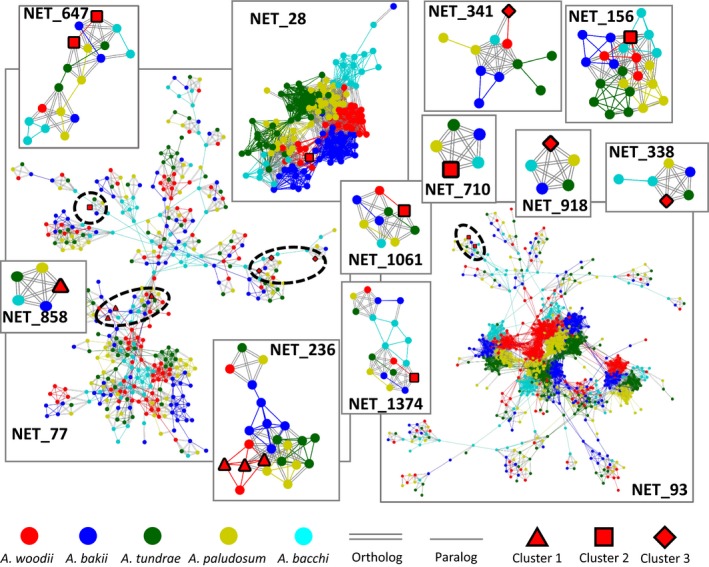
Extended version of Figure 7 showing the proteins of the three clusters of the WLP

We then selected the *A. woodii* proteins encoded by the genes of the WL pathway, identifying them within the generated networks. The proteins encoded by the gene clusters I, II, and III led to the discovery identification of 13 distinct networks (Figure A1). At least one protein per cluster presented cliques of one orthologue per genome (Figure 7), this is the case for FdhD in cluster I, FolD in cluster II and AcsD in cluster III (represented by NET_858, NET_710, and NET_918, respectively) (Figure 7). Gene expansion dynamics, represented as different numbers of paralogues occurring in different genomes, have been detected for a number of genes such as *fhs1* (Figure 4 and NET_341 of Figure 7), and *fchA* (NET_338 of Figure 7). More complex gene expansion dynamics were detected for the other genes (Figure A1). In particular, one out of three networks containing proteins encoded by the gene cluster I (NET_236), five out of eight networks (NET_28, NET_156, NET_647, NET_1061, and NET_1374) in cluster II, and one out of four networks containing proteins encoded by the gene cluster III (NET_341), display different numbers of duplicated genes within each network among all the other considered species. A few examples of specific trends regarding *A. bacchi* proteins are in NET_338, NET_647, and NET_1374, where *A. bacchi* orthologues are more numerous in comparison with the ones from the other species; in NET_341 and NET_1061 *A. bacchi* proteins are less common than the ones from the other species; in NET_236 *A. bacchi* proteins are completely missing (Figure A1). This confirms the divergence highlighted in the previous comparative analyses.

## 4 CONCLUSIONS

We obtained draft genome sequences for three *Acetobacterium* species and a acetogenic bacterium, *Alkalibaculum bacchi*. This study emphasizes the degree of genomic divergence and conservation of protein families within the genus*.* Having a closer look at the gene clusters involved in WL pathway, we revealed rearrangements and homology patterns that expands our understanding regarding the evolution of this metabolic pathway in the *Acetobacterium* genus with the perspective of future exploitation of these bacteria for industrial applications.

## CONFLICT OF INTERESTS

None declared.

## AUTHOR CONTRIBUTIONS

AE, ST, and OJ designed the study. AE, ST, LT, LA, and MLC analyzed and interpreted data. AE, ST, LA, and OJ wrote the manuscript. All authors read and approved the final manuscript.

## ETHICAL APPROVAL

None required.

## Data Availability

All data regarding this analysis were deposited in NCBI under the bioproject https://www.ncbi.nlm.nih.gov/bioproject/PRJNA509931

## APPENDIX 1

**Table A1:** Annotation of the genes in the core genome

RefSeq name in *A. woodii*	Cluster number	Gene name	*A. woodii*				*A. bacchi*			
			Contig	Start	End	Length	Contig	Start	End	Length
WP_014355214.1	1	fdhF1	NC_016894.1	944951	947125	2174	NODE_17_length_58697_cov_40.1842	55126	57810	2684
WP_014355215.1	1	hycB1	NC_016894.1	947122	947655	533	not found			
WP_014355216.1	1	fdhF2	NC_016894.1	947921	950089	2168	NODE_29_length_7652_cov_43.4377	4056	6758	2702
WP_014355217.1	1	hycB2	NC_016894.1	950093	950623	530	not found			
WP_083837833.1	1	fdhD	NC_016894.1	950758	951549	791	NODE_17_length_58697_cov_40.1842	50333	51133	800
WP_014355219.1	1	hycB3	NC_016894.1	951566	952126	560	not found			
WP_014355220.1	1	hydA1	NC_016894.1	952144	953523	1379	not found			
WP_014355320.1	2	fhs1	NC_016894.1	1080969	1082645	1676	NODE_3_length_279548_cov_33.281	195911	197584	1673
WP_014355321.1	2	fchA	NC_016894.1	1082745	1083404	659	NODE_3_length_279548_cov_33.281	197704	198330	626
WP_014355322.1	2	folD	NC_016894.1	1083442	1084347	905	NODE_3_length_279548_cov_33.281	198346	199197	851
WP_014355323.1	2	rnfC2	NC_016894.1	1084375	1086339	1964	NODE_7_length_185859_cov_36.1889	108899	110863	1964
WP_014355324.1	2	metV	NC_016894.1	1086341	1086958	617	NODE_7_length_185859_cov_36.1889	108265	108897	632
WP_014355325.1	2	metF	NC_016894.1	1086992	1087888	896	NODE_7_length_185859_cov_36.1889	107312	108193	881
WP_014355456.1	3	cooC1	NC_016894.1	1235110	1235895	785	NODE_3_length_279548_cov_33.281	182407	183177	770
WP_014355457.1	3	acsV	NC_016894.1	1235961	1237886	1925	NODE_3_length_279548_cov_33.281	187232	188480	1248
WP_014355458.1	3	orf1	NC_016894.1	1237902	1238549	647	not found			
WP_014355459.1	3	orf2	NC_016894.1	1238546	1239205	659	not found			
WP_014355460.1	3	acsD	NC_016894.1	1239392	1240327	935	NODE_3_length_279548_cov_33.281	183192	184139	947
WP_014355461.1	3	acsC	NC_016894.1	1240347	1241687	1340	NODE_3_length_279548_cov_33.281	184168	185508	1340
WP_014355462.1	3	acsE	NC_016894.1	1241757	1242542	785	NODE_3_length_279548_cov_33.281	185552	186337	785
WP_014355463.1	3	acsA	NC_016894.1	1242813	1244711	1898	NODE_3_length_279548_cov_33.282	177291	179183	1892
WP_014355464.1	3	cooC2	NC_016894.1	1244738	1245523	785	NODE_3_length_279548_cov_33.282	179205	179794	589
WP_041670690.1	3	acsB1	NC_016894.1	1245585	1247753	2168	NODE_3_length_279548_cov_33.282	180358	182149	1791

**Table A2:** Genomic coordinates of the WL pathway genes in *A. woodii* in comparison with *A. bacchi*

Gene name	Annotation
ackA	Acetate kinase
acoA	"Acetoin:2,6‐dichlorophenolindophenol oxidoreductase subunit alpha"
acsC	Corrinoid/iron‐sulfur protein large subunit
acsE	5‐methyltetrahydrofolate:corrinoid/iron‐sulfur protein co‐methyltransferase
alaA	Glutamate‐pyruvate aminotransferase AlaA
alaS	Alanine‐‐tRNA ligase
apbC	Iron‐sulfur cluster carrier protein
apeA	putative M18 family aminopeptidase 1
arcB	"Ornithine carbamoyltransferase 2, catabolic"
argC	N‐acetyl‐gamma‐glutamyl‐phosphate reductase
argD	acetylornithine aminotransferase ArgD1
argG	Argininosuccinate synthase
argH	Argininosuccinate lyase
argS	Arginine‐‐tRNA ligase
artM	Arginine transport ATP‐binding protein ArtM
asd2	Aspartate‐semialdehyde dehydrogenase 2
aspS	Aspartate‐‐tRNA ligase
asrA	Anaerobic sulfite reductase subunit A
asrB	Anaerobic sulfite reductase subunit B
asrC	Anaerobic sulfite reductase subunit C
atpA	ATP synthase subunit alpha
atpB	ATP synthase subunit a
atpD	"ATP synthase subunit beta, sodium ion specific"
azr	FMN‐dependent NADPH‐azoreductase
bfmB	Methoxymalonate biosynthesis protein
carE	Caffeyl‐CoA reductase‐Etf complex subunit CarE
cbiF	Cobalt‐precorrin‐4 C(11)‐methyltransferase
cbiH	putative cobalt‐factor III C(17)‐methyltransferase
cfiB	2‐oxoglutarate carboxylase small subunit
cheY	Chemotaxis protein CheY
clpP	ATP‐dependent Clp protease proteolytic subunit
clpX	ATP‐dependent Clp protease ATP‐binding subunit ClpX
clpY	ATP‐dependent protease ATPase subunit ClpY
coaX	Type III pantothenate kinase
cooS1	Carbon monoxide dehydrogenase 1
crh	HPr‐like protein Crh
csd	putative cysteine desulfurase
cysK1	O‐acetylserine sulfhydrylase
cysS	Cysteine‐‐tRNA ligase
dcd	dCTP deaminase
ddpD	putative D%2CD‐dipeptide transport ATP‐binding protein DdpD
der	GTPase Der
dmdA	2%2C3‐dimethylmalate dehydratase large subunit
dnaA	Chromosomal replication initiator protein DnaA
dnaE	DNA polymerase III subunit alpha
drrA	Daunorubicin/doxorubicin resistance ATP‐binding protein DrrA
dtd	D‐aminoacyl‐tRNA deacylase
dut	Deoxyuridine 5'‐triphosphate nucleotidohydrolase
dxs	1‐deoxy‐D‐xylulose‐5‐phosphate synthase
ecfA1	Energy‐coupling factor transporter ATP‐binding protein EcfA1
ecfA2	Energy‐coupling factor transporter ATP‐binding protein EcfA2
ecfT	Energy‐coupling factor transporter transmembrane protein EcfT
ecsA	ABC‐type transporter ATP‐binding protein EcsA
efp	Elongation factor P
eno	Enolase
era	GTPase Era
fba	Fructose‐bisphosphate aldolase
fbp	Fructose‐1%2C6‐bisphosphatase class 3
fchA	Methenyltetrahydrofolate cyclohydrolase
ffh	Signal recognition particle protein
fom3	2‐hydroxyethylphosphonate methyltransferase
frr	Ribosome‐recycling factor
ftsH	ATP‐dependent zinc metalloprotease FtsH
ftsZ	Cell division protein FtsZ
fumA	Fumarate hydratase class I%2C aerobic
fusA	Elongation factor G
gap	Glyceraldehyde‐3‐phosphate dehydrogenase
gatA	Glutamyl‐tRNA(Gln) amidotransferase subunit A
gatB	Aspartyl/glutamyl‐tRNA(Asn/Gln) amidotransferase subunit B
gatC	Aspartyl/glutamyl‐tRNA(Asn/Gln) amidotransferase subunit C
glmM	Phosphoglucosamine mutase
glmS	Glutamine‐‐fructose‐6‐phosphate aminotransferase [isomerizing]
glnH	Glutamine‐binding periplasmic protein
glnS	Glutamine‐‐tRNA ligase
glpK	Glycerol kinase
gltB	Ferredoxin‐dependent glutamate synthase 1
gltD	Glutamate synthase [NADPH] small chain
glyA	Serine hydroxymethyltransferase
glyQS	Glycine‐‐tRNA ligase
gmk	Guanylate kinase
gpmI	2%2C3‐bisphosphoglycerate‐independent phosphoglycerate mutase
graR	Response regulator protein GraR
groS	10 kDa chaperonin
gtaB	UTP‐‐glucose‐1‐phosphate uridylyltransferase
guaA	GMP synthase [glutamine‐hydrolyzing]
guaB	Inosine‐5'‐monophosphate dehydrogenase
gyrA	DNA gyrase subunit A
gyrB	DNA gyrase subunit B
hadI	2‐hydroxyisocaproyl‐CoA dehydratase activator
hcp	Hydroxylamine reductase
hemL	Glutamate‐1‐semialdehyde 2%2C1‐aminomutase
hicd	Homoisocitrate dehydrogenase
hinT	Purine nucleoside phosphoramidase
hisD	Histidinol dehydrogenase
hisF	Imidazole glycerol phosphate synthase subunit HisF
hisG	ATP phosphoribosyltransferase
hisH	Imidazole glycerol phosphate synthase subunit HisH
hisI	Phosphoribosyl‐AMP cyclohydrolase
hrb	High molecular weight rubredoxin
hslR	Heat shock protein 15
hslV	ATP‐dependent protease subunit HslV
htpG	Chaperone protein HtpG
hup	DNA‐binding protein HU
ileS	Isoleucine‐‐tRNA ligase
ilvB	Acetolactate synthase large subunit
ilvC	Ketol‐acid reductoisomerase (NADP(+))
ilvD	Dihydroxy‐acid dehydratase
ilvH	Putative acetolactate synthase small subunit
ilvK	Branched‐chain‐amino‐acid aminotransferase 2
infA	Translation initiation factor IF‐1
infC	Translation initiation factor IF‐3
iscS	Cysteine desulfurase IscS
iscU	Iron‐sulfur cluster assembly scaffold protein IscU
ispF	2‐C‐methyl‐D‐erythritol 2%2C4‐cyclodiphosphate synthase
ispG	4‐hydroxy‐3‐methylbut‐2‐en‐1‐yl diphosphate synthase (flavodoxin)
lepA	Elongation factor 4
leuB	3‐isopropylmalate dehydrogenase
leuD1	3‐isopropylmalate dehydratase small subunit 1
leuS	Leucine‐‐tRNA ligase
livF	High‐affinity branched‐chain amino acid transport ATP‐binding protein LivF
livH	High‐affinity branched‐chain amino acid transport system permease protein LivH
lon1	Lon protease 1
lptB	Lipopolysaccharide export system ATP‐binding protein LptB
lysC	Aspartokinase
lysS	Lysine‐‐tRNA ligase
map	Methionine aminopeptidase 1
metA	Homoserine O‐succinyltransferase
metG	Methionine‐‐tRNA ligase
metH	Methionine synthase
metI	D‐methionine transport system permease protein MetI
metN	Methionine import ATP‐binding protein MetN
metQ	Methionine‐binding lipoprotein MetQ
mgl	L‐methionine gamma‐lyase
miaB	tRNA‐2‐methylthio‐N(6)‐dimethylallyladenosine synthase
minD	Septum site‐determining protein MinD
mnmA	tRNA‐specific 2‐thiouridylase MnmA
mnmG	tRNA uridine 5‐carboxymethylaminomethyl modification enzyme MnmG
mog	Molybdopterin adenylyltransferase
mop	Aldehyde oxidoreductase
mprA	Response regulator MprA
mraZ	Transcriptional regulator MraZ
murAB	UDP‐N‐acetylglucosamine 1‐carboxyvinyltransferase 2
nikB	Nickel transport system permease protein NikB
nrdD	Anaerobic ribonucleoside‐triphosphate reductase
nrdJ	Vitamin B12‐dependent ribonucleotide reductase
nrdR	Transcriptional repressor NrdR
nspC	Carboxynorspermidine/carboxyspermidine decarboxylase
nth	Endonuclease III
ntpB	V‐type sodium ATPase subunit B
nusA	Transcription termination/antitermination protein NusA
nusG	Transcription termination/antitermination protein NusG
obg	GTPase Obg
oppF	Oligopeptide transport ATP‐binding protein OppF
paaK	Phenylacetate‐coenzyme A ligase
pduL	Phosphate propanoyltransferase
pfkA	ATP‐dependent 6‐phosphofructokinase
pgk	Phosphoglycerate kinase
pgsA	CDP‐diacylglycerol‐‐glycerol‐3‐phosphate 3‐phosphatidyltransferase
pheS	Phenylalanine‐‐tRNA ligase alpha subunit
pmpR	Transcriptional regulatory protein PmpR
pncB2	Nicotinate phosphoribosyltransferase pncB2
pnp	Polyribonucleotide nucleotidyltransferase
ppdK	Pyruvate%2C phosphate dikinase
ppiB	Peptidyl‐prolyl cis‐trans isomerase B
prfA	Peptide chain release factor 1
prfB	Peptide chain release factor 2
proA	Gamma‐glutamyl phosphate reductase
proS	Proline‐‐tRNA ligase
prs	Ribose‐phosphate pyrophosphokinase
pstB3	Phosphate import ATP‐binding protein PstB 3
pstC	Phosphate transport system permease protein PstC
pstS	Phosphate‐binding protein PstS
ptsI	Phosphoenolpyruvate‐protein phosphotransferase
purC	Phosphoribosylaminoimidazole‐succinocarboxamide synthase
purD	Phosphoribosylamine‐‐glycine ligase
purE	N5‐carboxyaminoimidazole ribonucleotide mutase
purF	Amidophosphoribosyltransferase
purH	Bifunctional purine biosynthesis protein PurH
purU	Formyltetrahydrofolate deformylase
pyrB	Aspartate carbamoyltransferase catalytic subunit
pyrD	Dihydroorotate dehydrogenase B (NAD(+))%2C catalytic subunit
pyrE	Orotate phosphoribosyltransferase
pyrF	Orotidine 5'‐phosphate decarboxylase
pyrG	CTP synthase
pyrH	Uridylate kinase
pyrI	Aspartate carbamoyltransferase regulatory chain
queA	S‐adenosylmethionine:tRNA ribosyltransferase‐isomerase
rarA	Replication‐associated recombination protein A
recA	Protein RecA
recU	Holliday junction resolvase RecU
rffG	dTDP‐glucose 4%2C6‐dehydratase 2
rhlE	ATP‐dependent RNA helicase RhlE
rho	Transcription termination factor Rho
ribH	6%2C7‐dimethyl‐8‐ribityllumazine synthase
rlmH	Ribosomal RNA large subunit methyltransferase H
rlmL	Ribosomal RNA large subunit methyltransferase K/L
rmlA	Glucose‐1‐phosphate thymidylyltransferase
rnfC	Electron transport complex subunit RnfC
rnfE	Electron transport complex subunit RnfE
rnhA	Ribonuclease H
rnjA	Ribonuclease J1
rny	Ribonuclease Y
rph	Ribonuclease PH
rplA	50S ribosomal protein L1
rplB	50S ribosomal protein L2
rplC	50S ribosomal protein L3
rplD	50S ribosomal protein L4
rplE	50S ribosomal protein L5
rplF	50S ribosomal protein L6
rplJ	50S ribosomal protein L10
rplK	50S ribosomal protein L11
rplL	50S ribosomal protein L7/L12
rplM	50S ribosomal protein L13
rplN	50S ribosomal protein L14
rplO	50S ribosomal protein L15
rplP	50S ribosomal protein L16
rplQ	50S ribosomal protein L17
rplR	50S ribosomal protein L18
rplS	50S ribosomal protein L19
rplT	50S ribosomal protein L20
rplU	50S ribosomal protein L21
rplV	50S ribosomal protein L22
rplW	50S ribosomal protein L23
rplX	50S ribosomal protein L24
rpmA	50S ribosomal protein L27
rpmB	50S ribosomal protein L28
rpmC	50S ribosomal protein L29
rpmD	50S ribosomal protein L30
rpmE	50S ribosomal protein L31
rpmF	50S ribosomal protein L32
rpmG	50S ribosomal protein L33
rpmI	50S ribosomal protein L35
rpoA	DNA‐directed RNA polymerase subunit alpha
rpoB	DNA‐directed RNA polymerase subunit beta
rpoC	DNA‐directed RNA polymerase subunit beta'
rpoZ	DNA‐directed RNA polymerase subunit omega
rpsB	30S ribosomal protein S2
rpsC	30S ribosomal protein S3
rpsD	30S ribosomal protein S4
rpsE	30S ribosomal protein S5
rpsF	30S ribosomal protein S6
rpsG	30S ribosomal protein S7
rpsH	30S ribosomal protein S8
rpsI	30S ribosomal protein S9
rpsJ	30S ribosomal protein S10
rpsK	30S ribosomal protein S11
rpsL	30S ribosomal protein S12
rpsM	30S ribosomal protein S13
rpsO	30S ribosomal protein S15
rpsP	30S ribosomal protein S16
rpsQ	30S ribosomal protein S17
rpsR	30S ribosomal protein S18
rpsS	30S ribosomal protein S19
rpsT	30S ribosomal protein S20
rpsU	30S ribosomal protein S21
rsfS	Ribosomal silencing factor RsfS
rsmH	Ribosomal RNA small subunit methyltransferase H
rsxA	Electron transport complex subunit RsxA
rsxB	Electron transport complex subunit RsxB
rsxD	Electron transport complex subunit RsxD
ruvB	Holliday junction ATP‐dependent DNA helicase RuvB
sbcD	Nuclease SbcCD subunit D
secA	Protein translocase subunit SecA
secY	Protein translocase subunit SecY
serC	Phosphoserine aminotransferase
serS	Serine‐‐tRNA ligase
sigA	RNA polymerase sigma factor SigA
smpB	SsrA‐binding protein
soj	Sporulation initiation inhibitor protein Soj
speA	Arginine decarboxylase
speB	Agmatinase
speD	S‐adenosylmethionine decarboxylase proenzyme
speE	Polyamine aminopropyltransferase
spoIIIE	DNA translocase SpoIIIE
spoVG	Putative septation protein SpoVG
sucB	Dihydrolipoyllysine‐residue succinyltransferase component of 2‐oxoglutarate dehydrogenase complex
tdcB	L‐threonine ammonia‐lyase
tgt	Queuine tRNA‐ribosyltransferase
thiC	Phosphomethylpyrimidine synthase
thiD	Hydroxymethylpyrimidine/phosphomethylpyrimidine kinase
thiH	2‐iminoacetate synthase
thiM	Hydroxyethylthiazole kinase
thiQ	Thiamine import ATP‐binding protein ThiQ
thrZ	Threonine‐‐tRNA ligase 2
thyX	Flavin‐dependent thymidylate synthase
tktA	Transketolase 1
trmL	tRNA (cytidine(34)‐2'‐O)‐methyltransferase
trpB	Tryptophan synthase beta chain
trpS	Tryptophan‐‐tRNA ligase
tsf	Elongation factor Ts
typA	GTP‐binding protein TypA/BipA
tyrS	Tyrosine‐‐tRNA ligase
ung	Uracil‐DNA glycosylase
upp	Uracil phosphoribosyltransferase
uppP	Undecaprenyl‐diphosphatase
uvrA	UvrABC system protein A
uvrB	UvrABC system protein B
valS	Valine‐‐tRNA ligase
walR	Transcriptional regulatory protein WalR
xpt	Xanthine phosphoribosyltransferase
ybiT	putative ABC transporter ATP‐binding protein YbiT
ychF	Ribosome‐binding ATPase YchF
ydcP	putative protease YdcP
yitJ	Bifunctional homocysteine S‐methyltransferase/5%2C10‐methylenetetrahydrofolate reductase
yknY	putative ABC transporter ATP‐binding protein YknY
yrrK	Putative pre‐16S rRNA nuclease
yxdL	ABC transporter ATP‐binding protein YxdL
	

## References

[mbo3938-bib-0001] Adam, P. S. , Borrel, G. , & Gribaldo, S. (2018). Evolutionary history of carbon monoxide dehydrogenase/acetyl‐CoA synthase, one of the oldest enzymatic complexes. Proceedings of the National Academy of Sciences, 115(6), E1166–E1173. 10.1073/pnas.1716667115 PMC5819426 29358391

[mbo3938-bib-0002] Ambrosino, L. , Ruggieri, V. , Bostan, H. , Miralto, M. , Vitulo, N. , Zouine, M. , … Valle, G. (2018). Multilevel comparative bioinformatics to investigate evolutionary relationships and specificities in gene annotations. BMC Bioinformatics, 19(15), 435 10.1186/s12859-018-2420-y 30497367 PMC6266932

[mbo3938-bib-0003] Andrews, S. FastQC: A quality control tool for high throughput sequence data. 2010 Retrieved from http://www.bioinformatics.babraham.ac.uk/projects/. doi:citeulike‐article‐id:11583827.

[mbo3938-bib-0004] Balch, W. E. , Schoberth, S. , Tanner, R. S. , & Wolfe, R. S. (1977). Acetobacterium, a new genus of hydrogen‐oxidizing, carbon dioxide‐reducing, anaerobic Bacteria. International Journal of Systematic Bacteriology, 27(4), 355–361. 10.1099/00207713-27-4-355

[mbo3938-bib-0005] Bankevich, A. , Nurk, S. , Antipov, D. , Gurevich, A. A. , Dvorkin, M. , Kulikov, A. S. , … Pyshkin, A. V. (2012). SPAdes: A new genome assembly algorithm and its applications to single‐cell sequencing. Journal of Computational Biology, 19(5), 455–477. 10.1089/cmb.2012.0021 22506599 PMC3342519

[mbo3938-bib-0006] Bengelsdorf, F. R. , Poehlein, A. , Linder, S. , Erz, C. , Hummel, T. , Hoffmeister, S. , … Dürre, P. (2016). Industrial acetogenic biocatalysts: A comparative metabolic and genomic analysis. Frontiers in Microbiology, 7, 1036 10.3389/fmicb.2016.01036 27458439 PMC4935695

[mbo3938-bib-0007] Berg, I. A. (2011). Ecological aspects of the distribution of different autotrophic CO2 fixation pathways. Applied and Environment Microbiology, 77(6), 1925–1936. 10.1128/AEM.02473-10 PMC3067309 21216907

[mbo3938-bib-0008] Blin, K. , Wolf, T. , Chevrette, M. G. , Lu, X. , Schwalen, C. J. , Kautsar, S. A. , … Dickschat, J. S. (2017). AntiSMASH 4.0 ‐ improvements in chemistry prediction and gene cluster boundary identification. Nucleic Acids Research, 45(W1), W36–W41. 10.1093/nar/gkx319 28460038 PMC5570095

[mbo3938-bib-0009] Borrel, G. , Adam, P. S. , & Gribaldo, S. (2016). Methanogenesis and the Wood‐Ljungdahl Pathway: An ancient, verstatile, and fragile association. Genome Biology and Evolution, 8(6), 1706–1711. 10.1093/gbe/evw114 27189979 PMC4943185

[mbo3938-bib-0010] Bratlie, M. S. , Johansen, J. , Sherman, B. T. , Huang, D. W. , Lempicki, R. A. , & Drablos, F. (2010). Gene duplications in prokaryotes can be associated with environmental adaptation. BMC Genomics, 11(1), 588 10.1186/1471-2164-11-588 20961426 PMC3091735

[mbo3938-bib-0011] Camacho, C. , Coulouris, G. , Avagyan, V. , Ma, N. , Papadopoulos, J. , Bealer, K. , Madden, T. L. (2009). BLAST+: Architecture and applications. BMC Bioinformatics, 10(1), 421 10.1186/1471-2105-10-421 20003500 PMC2803857

[mbo3938-bib-0012] Cavicchioli, R. , Charlton, T. , Ertan, H. , Omar, S. M. , Siddiqui, K. S. , & Williams, T. J. (2011). Biotechnological uses of enzymes from psychrophiles. Microbial Biotechnology, 4(4), 449–460. 10.1111/j.1751-7915.2011.00258.x 21733127 PMC3815257

[mbo3938-bib-0013] Drake, H. L. (1994). Acetogenesis (1st ed.). US: Springer 10.1007/978-1-4615-1777-1

[mbo3938-bib-0014] Graber, J. R. , & Breznak, J. A. (2004). Physiology and nutrition of Treponema primitia, an H2/CO2‐acetogenic spirochete from termite hindguts. Applied and Environment Microbiology, 70(3), 1307–1314. 10.1128/AEM.70.3.1307-1314.2004 PMC368360 15006747

[mbo3938-bib-0015] Guy, L. , Kultima, J. R. , Andersson, S. G. E. , & Quackenbush, J. (2011). GenoPlotR: Comparative gene and genome visualization in R. Bioinformatics, 26(18), 2334–2335. 10.1093/bioinformatics/btq413 PMC2935412 20624783

[mbo3938-bib-0016] Hagberg, A. A. , Schult, D. A. , & Swart, P. J. (2008). Exploring network structure, dynamics, and function using NetworkX. Proc 7th. Python Sci Conf., 10.1016/j.jelectrocard.2010.09.003

[mbo3938-bib-0017] Huerta‐Cepas, J. , Forslund, K. , Coelho, L. P. , Szklarczyk, D. , Jensen, L. J. , Von Mering, C. , Bork, P. (2017). Fast genome‐wide functional annotation through orthology assignment by eggNOG‐mapper. Molecular Biology and Evolution, 34(8), 2115–2122. 10.1093/molbev/msx148 28460117 PMC5850834

[mbo3938-bib-0018] Hug, L. A. , Castelle, C. J. , Wrighton, K. C. , Thomas, B. C. , Sharon, I. , Frischkorn, K. R. , … Banfield, J. F. (2013). Community genomic analyses constrain the distribution of metabolic traits across the Chloroflexi phylum and indicate roles in sediment carbon cycling. Microbiome, 1(1), 22 10.1186/2049-2618-1-22 24450983 PMC3971608

[mbo3938-bib-0019] Hughes, A. L. (2005). Gene duplication and the origin of novel proteins. Proceedings of the National Academy of Sciences, 102(25), 8791–8792. 10.1073/pnas.0503922102 PMC1157061 15956198

[mbo3938-bib-0020] Huynen, M. A. , & Bork, P. (1998). Measuring genome evolution. Proceedings of the National Academy of Sciences, 95(11), 5849–5856. 10.1073/pnas.95.11.5849 PMC34486 9600883

[mbo3938-bib-0021] Hwang, S. , Song, Y. , & Cho, B.‐K. (2015). Draft genome sequence of Acetobacterium bakii DSM 8239, a potential psychrophilic chemical producer through syngas fermentation. Genome Announcements, 3(5), e01070–e1115. 10.1128/genomeA.01070-15 26404601 PMC4582577

[mbo3938-bib-0022] Kondrashov, F. A. (2012). Gene duplication as a mechanism of genomic adaptation to a changing environment. Proceedings of the Royal Society B: Biological Sciences, 279(1749), 5048–5057. 10.1098/rspb.2012.1108 PMC3497230 22977152

[mbo3938-bib-0023] Krueger, F. (2016). Trim Galore. In: Babraham Bioinformatics, Retrieved from http://www.bioinformatics.babraham.ac.uk/projects/trimgalore/

[mbo3938-bib-0024] Overbeek, R. , Fonstein, M. , D'Souza, M. , Pusch, G. D. , & Maltsev, N. (1999). The use of gene clusters to infer functional coupling. Proceedings of the National Academy of Sciences, 96(6), 2896–2901. 10.1073/pnas.96.6.2896 PMC15866 10077608

[mbo3938-bib-0025] Page, A. J. , Cummins, C. A. , Hunt, M. , Wong, V. K. , Reuter, S. , Holden, M. T. G. , … Parkhill, J. (2015). Roary: Rapid large‐scale prokaryote pan genome analysis. Bioinformatics, 31(22), 3691–3693. 10.1093/bioinformatics/btv421 26198102 PMC4817141

[mbo3938-bib-0026] Poehlein, A. , Schmidt, S. , Kaster, A. K. , Goenrich, M. , Vollmers, J. , Thürmer, A. , … Müller, V. (2012). An ancient pathway combining carbon dioxide fixation with the generation and utilization of a sodium ion gradient for ATP synthesis. PLoS ONE, 7(3), e33439 10.1371/journal.pone.0033439 22479398 PMC3315566

[mbo3938-bib-0027] Pritchard, L. , Glover, R. H. , Humphris, S. , Elphinstone, J. G. , & Toth, I. K. (2016). Genomics and taxonomy in diagnostics for food security: Soft‐rotting enterobacterial plant pathogens. Analytical Methods, 8(1), 12–24. 10.1039/C5AY02550H

[mbo3938-bib-0028] R Core Team . R: A Language and Environment for Statistical Computing. Vienna, Austria; 2012 Retrieved from http://www.r-project.org

[mbo3938-bib-0029] Ragsdale, S. W. , & Pierce, E. (2008). Acetogenesis and the Wood‐Ljungdahl pathway of CO2 fixation. Biochimica Et Biophysica Acta (BBA)‐Proteins and Proteomics, 1784(12), 1873–1898. 10.1016/j.bbapap.2008.08.012 18801467 PMC2646786

[mbo3938-bib-0030] Rosenfeld, J. A. , & DeSalle, R. (2012). E value cutoff and eukaryotic genome content phylogenetics. Molecular Phylogenetics and Evolution, 63(2), 342–350. 10.1016/j.ympev.2012.01.003 22306824

[mbo3938-bib-0031] Schuchmann, K. , & Mueller, V. (2014). Autotrophy at the thermodynamic limit of life: A model for energy conservation in acetogenic bacteria. Nature Reviews Microbiology, 12(12), 809 10.1038/nrmicro3365 25383604

[mbo3938-bib-0032] Schuchmann, K. , & Mueller, V. (2016). Energetics and application of heterotrophy in acetogenic bacteria. Applied and Environment Microbiology, 82(14), 4056–4069. 10.1128/AEM.00882-16 PMC4959221 27208103

[mbo3938-bib-0033] Seemann, T. (2014). Prokka: Rapid prokaryotic genome annotation. Bioinformatics, 30(14), 2068–2069. 10.1093/bioinformatics/btu153 24642063

[mbo3938-bib-0034] Shannon, P. , Markiel, A. , Ozier, O. , Baliga, N. S. , Wang, J. T. , Ramage, D. , et al. (2003). Cytoscape: A software Environment for integrated models of biomolecular interaction networks. Genome Research, 13(11), 2498–2504. 10.1101/gr.1239303 14597658 PMC403769

[mbo3938-bib-0035] Shin, J. , Song, Y. , Jeong, Y. , & Cho, B. K. (2016). Analysis of the core genome and pan‐genome of autotrophic acetogenic bacteria. Frontiers in Microbiology, 7, 1531 10.3389/fmicb.2016.01531 27733845 PMC5039349

[mbo3938-bib-0036] Shin, J. , Song, Y. , Jin, S. , Lee, J.‐K. , Kim, D. R. , Kim, S. C. , … Cho, B. K. (2018). Genome‐scale analysis of Acetobacterium bakii reveals the cold adaptation of psychrotolerant acetogens by post‐transcriptional regulation. RNA, 24(12), 1839–1855. 10.1261/rna.068239.118 30249742 PMC6239172

[mbo3938-bib-0037] Simankova, M. V. , Kotsyurbenko, O. R. , Stackebrandt, E. , Kostrikina, N. A. , Lysenko, A. M. , Osipov, G. A. , Nozhevnikova, A. N. (2000). Acetobacterium tundrae sp.nov., a new psychrophilic acetogenic bacterium from tundra soil. Archives of Microbiology, 174(6), 440–447. 10.1007/s002030000229 11195100

[mbo3938-bib-0038] Strous, M. , Pelletier, E. , Mangenot, S. , Rattei, T. , Lehner, A. , Taylor, M. W. , … Barbe, V. (2006). Deciphering the evolution and metabolism of an annamox bacterium from a community genome. Nature, 440(7085), 790 10.1038/nature04647 16598256

[mbo3938-bib-0039] Tatusov, R. L. , Koonin, E. V. , & Lipman, D. J. (1997). A genomic perspective on protein families. Science, 278(5338), 631–637. 10.1126/science.278.5338.631 9381173

[mbo3938-bib-0040] Weiss, M. C. , Sousa, F. L. , Mrnjavac, N. , Neukirchen, S. , Roettger, M. , Nelson‐Sathi, S. , … Martin, W. F. (2016). The physiology and habitat of the last universal common ancestor. Nature Microbiology, 1(9), 16116 10.1038/NMICROBIOL.2016.116 27562259

